# Non-operative management attempted for selective high grade blunt hepatosplenic trauma is a feasible strategy

**DOI:** 10.1186/1749-7922-9-51

**Published:** 2014-09-25

**Authors:** Ting-Min Hsieh, Tsung Cheng Tsai, Jiun-Lung Liang, Chih Che Lin

**Affiliations:** Division of Trauma Surgery, Kaohsiung Chang Gung Memorial Hospital and Chang Gung University College of Medicine, 123 Ta Pei Road, Niao-Sung District, Kaohsiung, Taiwan; Department of Emergency, Kaohsiung Chang Gung Memorial Hospital and Chang Gung University College of Medicine, 123 Ta Pei Road, Niao-Sung District, Kaohsiung, Taiwan; Department of Radiology, Kaohsiung Chang Gung Memorial Hospital and Chang Gung University College of Medicine, 123 Ta Pei Road, Niao-Sung District, Kaohsiung, Taiwan; Division of General Surgery, Kaohsiung Chang Gung Memorial Hospital and Chang Gung University College of Medicine, 123 Ta Pei Road, Niao-Sung District, Kaohsiung, Taiwan

**Keywords:** Non-operative management, Blunt hepatic injury, Blunt splenic injury, Blunt hepatic and splenic injuries

## Abstract

**Background:**

There is growing evidence of clinical data recently for successful outcomes of non-operative management (NOM) for blunt hepatic and spleen injuries (BHSI). However, the effectiveness of NOM for high-grade BHSI remains undefined. The aim of the present study was to review our experience with NOM in high-grade BHSI and compare results with the existing related data worldwide.

**Methods:**

In this retrospectively protocol-driven study, 150 patients with grade 3–5 BHSI were enrolled during a 3-year period. Patients were divided into immediate laparotomy (immediate OP) and initial non-operative (initial NOM) groups according to hemodynamic status judged by duty trauma surgeon. Patients who received initial NOM were divided into successful NOM (s-NOM) and failed NOM (f-NOM) subgroups according to conservative treatment failure. We analyzed the clinical characteristics and the outcomes of patients.

**Results:**

Twenty-eight (18.7%) patients underwent immediate operations, and the remaining 122 (81.3%) were initially treated with NOM. Compared with the initial NOM group, the immediate OP group had significantly lower hemoglobin levels, a higher incidence of tube thoracostomy, contrast extravasation and large hemoperitoneum on computed tomography, a higher injury severity score, increased need for transfusions, and longer length of stay (LOS) in the intensive care unit (ICU) and hospitalization. Further analysis of the initial NOM group indicated that NOM had failed in 6 (4.9%) cases. Compared with the s-NOM subgroup, f-NOM patients had significantly lower hemoglobin levels, more hospitalized transfusions, and longer ICU LOS.

**Conclusions:**

NOM of high-grade BHSI in selected patients is a feasible strategy. Notwithstanding, patients with initial low hemoglobin level and a high number of blood transfusions in the ICU are associated with a high risk for NOM failure.

## Introduction

Blunt abdominal trauma (BAT) resulting from a traffic accident, fall, assault, or occupational accident is not unusual in the emergency room. The prevalence of intra-abdominal injury after BAT has been reported to be high at 12-15% [1]. The liver and spleen are the most commonly injured organs in BAT, accounting for up to 70% of all visceral injuries [2, 3]. Since the 1980s, there had been a paradigm shift from surgical to nonsurgical treatment for blunt hepatic and/or splenic injuries (BHSI). Many authors published their experiences showing satisfactory results [4, 5]. Computed tomography (CT), which can accurately assess the severity of organ injury, hemoperitoneum, presence of contrast extravasation, viscus injury, and can predict the necessity for prompt intervention, is the diagnostic modality of choice for hemodynamically stable patients. Routine follow-up CT is no longer suggested for NOM of patient with solid organ injury because it has poor ability to detect unidentified injuries [4–6]. Contrast-enhanced ultrasound (CEUS), which can provide a safe and accurate alternative to CT [7], and can guide a percutaneous treatment, is a save adjunct to observation in NOM [8]. An increasing body of literature emphasizing promising results, the wide use of CT, and the emergence of CEUS promoted the acceptance of non-operative management (NOM) as the standard therapeutic strategy. In fact, with numerous recent studies have shown success rates > 90% and failure rates < 11% [9–11]. Moreover, high success rates with NOM have been in pediatric patients [3]. Additionally, some studies [12, 13] have documented the feasibility and safety of NOM in patients with advanced age, or neurologic impairment, which were not recommended for NOM before. As the concept of NOM is now established, there is a growing concern regarding its morbidity and drawbacks of angioembolization, which are especially prevalent in high-grade injuries [14–18]. Moreover, the effectiveness of NOM in high-grade injuries is still under scrutiny. On the other hand, some authors suggested that surgeons should temper enthusiasm for NOM despite advances in the quality of critical care and radiological intervention [8, 19, 20]. Because few studies have focused exclusively on high-grade BHSI, the present study aimed to investigate the efficacy of NOM for complex BHSI in the setting of a tertiary care center.

## Methods

### Setting, study protocol

Patients admitted to Kaohsiung Chang Gung Memorial Hospital with BHSI between January 2010 to December 2012 were retrospectively reviewed. All patients were initially assessed and resuscitated at the emergency room (ER) according to the advanced trauma life support (ATLS) guidelines. The selection of patients for a nonsurgical management protocol [21] was based on the following criteria: hemodynamic stability on admission or shortly after initial resuscitation, maintenance of hemodynamic stability [systolic blood pressure (SBP) > 90 mmHg] without the need for excessive blood transfusion, no obvious peritonitis, and no associated multiple traumas requiring immediate operation. Indication signs for angiography were: significant hemoperitoneum (>1000 mL) with episode of hypotension (SBP < 90 mmHg) or contrast extravasation on CT scan, recurrent hypotension despite fluid resuscitation, grade 4–6 hepatic or grade 4–5 splenic injuries, and falling hemoglobin level (<8 g/dL) with progressive need for blood transfusions. We determined that angiography should be performed early after initial stabilization if the criteria were met. In the case of rapid clinical deterioration, the procedure was abandoned, and the patient underwent immediate emergency surgery.

### Data collection, definitions and exclusion criteria

Although this was a retrospective study, data on age, gender, mechanism of blunt trauma, initial vital signs [i.e. SBP, heart rate (HR), respiratory rate (RR)], hemoglobin level, Glasgow coma scale (GCS), alcohol intoxication, incidences of endotracheal intubation and tube thoracostomy, CT findings, Injury Severity Score (ISS), blood transfusion at ER and during admission, length of stay (LOS) at intensive care unit (ICU), duration of hospitalization, and outcomes, including morbidities and mortalities, were prospectively collected. ER transfusions included units of blood transfused during resuscitation at ER or before transfer from a local clinic, whereas admission transfusions referred to all units administered during hospitalization, except resuscitation at ER. The severity of BHSI was graded according to the classification of the American Association for the Surgery of Trauma (1994 revision). Patients with concomitant liver and spleen injuries were assigned to either liver or splenic injury group according to the organ with higher injury grading. The presence of intra-abdomen fluid was determined using CT. The amount of hemoperitoneum was quantified as follows: minimal, perihepatic blood in subphrenic or subhepatic space or perisplenic fossae (<500 mL); moderate, minimal plus blood along paracolic gutter (500-1000 mL); and large, moderate plus blood accumulating in pelvic cavity (>1000 mL). Patients who died at ER, those without available abdominal CT, and those with CT findings consistent with grade I or II injuries were excluded from the present study. High-grade injury referred grade III-VI in blunt hepatic injurIES (BHI) and grade III-V in blunt spleinc injuries (BSI).

### Study population and grouping

The patients were initially categorized into two groups: those initially treated non-operatively were included in the initial NOM group and those receiving early laparotomy at ER because of hemodynamic instability or suspected peritonitis were included in the immediate OP group. Patients in the initial NOM group were admitted to ICU for close monitoring and were further divided into two subgroups, the s-NOM included patients that treated successfully with conservative methods and the f-NOM included those who eventually required laparotomy according to the judgment of trauma surgeons after observation in ICU.

### Statistical analysis

Data are presented as percentages for categorical data, and means ± SE for ordinal and continuous data. Statistical analyses were performed using the chi-square test or Fisher’s exact test for discrete variables and the Mann Whitney U test for continuous variables. All differences at the p < 0.05 level were considered statistically significant.

## Results

### Patient characteristic, trauma mechanisms

During the 3-year study period, 150 patients presented with high-grade BHSI, of whom 91 and 59 had BHI and BSI, respectively. The relationship between the severity of hemoperitoneum and CT grading is shown in Table 1. The majority of the study subjects were men (62%), with a mean age of 31.9 ± 16.3 years (range, 3–77).

**Table 1:** **Correlations between severity of hemoperitoneum and grading of liver/spleen injuries on computed tomography**

Severity of hemoperitoneum	Grade of liver/spleen laceration					Total no. of patients
	I	II	III	IV	V	
Nil to minimal	2/3	49^(3)^/14	52^(2)^/17	12/0	4/1	119/35
Moderate	0/0	1/0	2/7^(1)^	4/1	1/2	8/10
Large	0/0	0/4	4^(1)^/11	7/10	5*/10^(2)^	16/35
Total	2/3	50/18	58/35	23/11	10/13	143/80

*Including a grade VI liver laceration with large hemoperitoneum, parentheses: means including patient number of concomitant liver and spleen injuries.

The most common causes of high-grade BHI were motorcycle collision (n = 55, 60.4%), motor vehicle collision (n = 18, 19.8%), falls from greater height (n = 7, 7.7%) or from own height (n = 4, 4.4%), pedestrian struck (n = 3, 3.3%), assaults (n = 2, 2.2%), and bicycle collision (n = 2, 2.2%). In high-grade BSI, motorcycle collisions were responsible for most injuries (n = 46, 78%), while other causes included motor vehicle collision (n = 4, 6.8%), assaults (n = 3, 5.1%), falls from own height (n = 2, 3.4%) and from greater height (n = 1, 1.7%), bicycle collision (n = 2, 3.4%), and pedestrian struck (n = 1, 1.7%) (Tables 2, 3 and 4).

**Table 2:** **Comparisons between initial NOM group and immediate OP group**

	Initial NOM	Immediate OP	***p***
Number of patients (n)	122	28	-
Gender (male)	73 (59.8%)	20 (71.4%)	0.25
Age (years)	32.52 ± 16.73	29.64 ± 14.47	0.40
SBP (mmHg)	118.68 ± 29.32	107.36 ± 28.85	0.06
HR (beats/min)	98.13 ± 20.31	105.11 ± 25.94	0.12
RR (breaths/min)	20.06 ± 3.65	21.82 ± 6.36	0.16
Hemoglobin (g/dL)	11.94 ± 2.34	10.46 ± 3.09	0.005
Endotracheal intubation (%)	13 (10.7%)	5 (17.9%)	0.33
Tube thoracostomy (%)	18 (14.8%)	10 (35.7%)	0.01
CT extravasation (%)	30 (24.6%)	16 (57.1%)	0.001
Large hemoperiotneum (%)	28 (23.0%)	19 (67.9%)	<0.001
Alcohol intoxication (%)	85 (69.7%)	24 (85.7%)	0.08
GCS	13.78 ± 2.73	13.11 ± 3.42	0.26
ISS	19.78 ± 10.35	26.30 ± 11.55	0.004
Mechanism:			0.45
Motorcycle	84 (69%)	17 (61%)	
Motor vehicle	15 (12%)	6 (21%)	
others	21 (19%)	5 (18%)	
Emergency room BT (U)	1.48 ± 2.05	5.14 ± 5.26	0.001
Hospitalization BT(U)	2.41 ± 4.98	10.86 ± 11.95	0.001
BT requirement (%)	73 (59.8%)	26 (92.9%)	0.001
Hospitalization LOS(day)	13.66 ± 10.20	21.64 ± 14.75	0.01
ICU LOS(day)	4.57 ± 4.45	8.68 ± 9.17	0.02
Patients with associated injury (%)	96 (78.7%)	23 (82.1%)	0.684
Patients with complication(s) (%)	14 (11.4%)	6 (21.4%)	0.12
Mortality (%)	6 (4.9%)	4 (14.3%)	0.09

NOM: Non-operative management; OP: Operation; SBP: Systolic blood pressure; CT: Computed tomography; GCS: Gasglow coma scale; ISS: Injury severity score; BT: Blood transfusion; ICU: Intensive care unit; LOS: Length of stay.

**Table 3:** **Comparisons between patients with s-NOM and f-NOM**

	Non-operative (s-NOM)	f-NOM	***P***
Number of patients (n)	116	6	-
Gender (male)	69 (59.5%)	4 (66.7%)	1.00
Age (years)	32.34 ± 16.21	36.00 ± 26.69	0.69
SBP (mmHg)	119.44 ± 29.37	104.00 ± 26.35	0.25
HR (beats/min)	98.13 ± 20.13	98.17 ± 25.66	0.74
RR (breaths/min)	20.03 ± 3.59	20.67 ± 5.00	0.95
Hemoglobin (g/dL)	12.11 ± 2.27	8.67 ± 0.51	0.001
Endotracheal intubation (%)	11 (9.5%)	2 (33.3%)	0.12
Tube thoracostomy (%)	16 (13.8%)	2 (33.3%)	0.21
CT extravasation (%)	29 (25.0%)	1 (16.7%)	1.00
Large hemoperiotneum (%)	25 (21.6%)	3 (50.0%)	0.13
Alcohol (%)	79 (68.1%)	6 (100.0%)	0.17
GCS	13.88 ± 2.55	11.83 ± 5.15	0.44
ISS	19.36 ± 10.24	27.83 ± 9.80	0.06
Mechanism:			0.47
Motorcycle	79 (68%)	5 (83%)	
Motor vehicle	14 (12%)	1 (17%)	
Others	23 (10%)	0 (0%)	
Emergency room BT (U)	1.43 ± 2.05	2.33 ± 1.96	0.12
Hospitalization BT (U)	2.01 ± 4.15	10.17 ± 11.35	0.001
BT requirement (%)	67 (57.8%)	6 (100.0%)	0.08
Hospitalization (days)	13.22 ± 9.28	22.17 ± 21.12	0.33
ICU LOS (days)	4.19 ± 3.60	12.00 ± 10.60	0.02
Patients with associated injury (%)	91 (78.4%)	5 (83.3%)	1.00
Patients with complication(s) (%)	8 (6.8%)	0 (0.0%)	1.00
Mortality (%)	4 (3.4%)	2 (33.3%)	0.02

**s**-NOM: Successful non-operative management; f-NOM: Failed non-operative management; SBP: Systolic blood pressure; CT: Computed tomography; GCS: Gasglow coma scale; ISS: Injury severity score; BT: Blood transfusion; ICU: Intensive care unit; LOS: Length of stay.

**Table 4:** **Comparisons between patients with and without operations for blunt high-grade liver or spleen injuries**

	Non-operative	Operative	***P***
	(s-NOM)	(f-NOM + Immediate OP)	
Number of patients (n)	116	34	-
Gender (male)	69 (59.5%)	24 (70.6%)	0.24
Age (years)	32.34 ± 16.21	30.76 ± 16.89	0.62
SBP (mmHg)	119.44 ± 29.37	106.76 ± 28.06	0.02
HR (beats/min)	98.13 ± 20.13	103.88 ± 25.64	0.23
RR (breaths/min)	20.03 ± 3.59	21.62 ± 6.09	0.15
Hemoglobin (g/dL)	12.11 ± 2.27	10.15 ± 2.89	<0.001
Endotracheal intubation (%)	11 (9.5%)	7 (20.6%)	0.12
Tube thoracostomy (%)	16 (13.8%)	12 (35.3%)	0.005
CT extravasation (%)	29 (25.0%)	17 (50.0%)	0.005
Large hemoperiotneum (%)	25 (21.6%)	22 (64.7%)	<0.001
Alcohol (%)	79 (68.1%)	30 (88.2%)	0.02
GCS	13.88 ± 2.55	12.88 ± 3.72	0.15
ISS	19.36 ± 10.24	26.58 ± 11.13	0.001
Mechanism:			0.41
Motorcycle	79 (68%)	22 (65%)	
Motor vehicle	14 (12%)	7 (21%)	
Others	23 (10%)	5 (15%)	
Emergency room BT (U)	1.43 ± 2.05	4.65 ± 4.94	0.001
Hospitalization BT (U)	2.01 ± 4.15	11.06 ± 11.70	<0.001
BT requirement (%)	67 (57.8%)	32 (94.1%)	<0.001
Hospitalization (days)	13.22 ± 9.28	21.74 ± 15.67	0.004
ICU LOS (days)	4.19 ± 3.60	9.26 ± 9.35	0.004
Patients with associated injury (%)	91 (78.4%)	28 (82.4%)	0.62
Patients with complication(s) (%)	8 (6.8%)	6 (17.6%)	0.27
Mortality (%)	4 (3.4%)	6 (17.6%)	0.01

**s**-NOM: Successful non-operative management; f-NOM: Failed non-operative management; OP: Operation; SBP: Systolic blood pressure; CT: Computed tomography; GCS: Gasglow coma scale; ISS: Injury severity score; BT: Blood transfusion; ICU: Intensive care unit; LOS: Length of stay.

### Patient management algorithm, final outcomes

**Figure 1:**
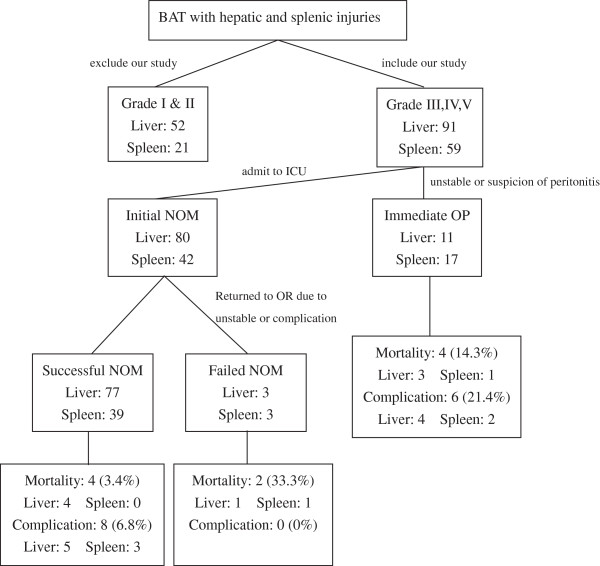
**NOM was initially applied in 81.3% of all patients with high-grade blunt hepatic and/or splenic injuries with a failure rate of 4.9%.** NOM: non-operative management; BAT: blunt abdominal trauma; ICU: intensive care unit; OR: operation room.

The patient population, morbidity, mortality, and management algorithm are described in Figure 1. The causes of failure of NOM included complications in 14 patients in the initial NOM group (11.4%, 14/122). The f-NOM group included 6 patients, and the s-NOM group included 8 patients. Of the 6 patients in the f-NOM, 1 presented with BSI with persistent hemorrhage and atrial fibrillation attack, 1 with a history of liver cirrhosis showing re-bleeding after splenic angioembolization, 1 had splenic abscess with profound sepsis after splenic angioembolization, 2 showed reduced hemoglobin levels despite active resuscitation and hepatic angioembolization, and 1 showed unstable hemodynamics with concomitant BHSI and lung contusion. Complications, including re-bleeding (n = 2), liver abscess (n = 2), empyema (n = 1), intra-abdominal abscess (n = 2), and intestinal obstruction (n = 1), were successfully treated conservatively in the remaining 8 patients in the s-NOM group. Six patients in the immediate OP group developed complications, including sepsis (n = 1), the formation of intra-abdominal abscess (n = 3), hepatic abscess (n = 1), and biloma (n = 1), which were also successfully treated conservatively.

In addition, there were 10 deaths, including 4 in the s-NOM group, 2 in the f-NOM group, and 4 in the immediate OP group. Of the 4 patients in the s-NOM group, 3 died of intracranial hemorrhage and 1 died of severe lung contusion. Of the 4 patients in the immediate OP, 1 with grade V BHI died of persistent shock postoperatively, 1 with BHI and pelvis fracture died of massive transfusion-related coagulopathy, 1 with BHI and mesentery tear died of liver cirrhosis, and 1 with BSI died of intracranial hemorrhage. Of the 2 patients in the f-NOM group, 1 with concomitant BHSI died of severe lung contusion (ISS:34) on the second postoperative day, and 1 with grade IV BSI (ISS:38) and post angioembolization re-bleeding died of liver cirrhosis 6 days postoperatively.

### Initial NOM vs. immediate OP

NOM was initially applied in 81.3% (n = 122) of all patients with high-grade BHSI. Twenty-eight (18.7%) patients underwent emergency laparotomy. The incidences of initial NOM for high-grade BHSI were 88% (80/91) and 71% (42/59), respectively. The comparisons of characteristics of the initial NOM and immediate OP groups are presented in Table 2.

### s-NOM vs. f-NOM

Of the 122 patients initially treated with NOM, 116 were treated successfully (95%). Further analysis of the two subgroups of the initial NOM group is presented in Table 3.

### Non-operative vs. patients receiving operations

In terms of operative treatment, comparisons between the s-NOM and patients receiving operations (immediate OP + f-NOM) are shown in Table 4.

## Discussion

NOM is currently the main treatment for patients with BHSI and has shown excellent results [4, 5, 8, 11, 13–15]. This may be partly attributed to the aggressive use of angioembolization in recent years [14, 15, 17, 21, 22]. Another factor is that strict use of a protocol based approach and algorithm leads to a significantly expansion of NOM. According to the study of Miller et al. [14], the failure rate of NOM attempted for high-grade BSI improved from 15% to 5% with the incorporation of a protocol. Mitsusada et al. [23] reported that NOM of BHI applied for selected hemodynamically unstable patients (target SBP of 80 mmHg) under a revision protocol can decrease the overall laparotomy rates and transfusion requirements. Accordingly, a protocol based algorithm for the management of BHSI is proposed.

In present study, NOM was applied in 81.3% of high-grade BHSI patients with a failure rate of 3.7% and 7.1% for BHI and BSI, respectively, resulting in an overall failure rate of 4.9%. Our results are comparable to those of prior studies [8, 9] showing that 72%-81% of BHSI patients are treated by NOM with a failure rate of 5.2%-5.8%. On the other hand, unlike previous studies [8, 9], the present study focused exclusively on high-grade injuries. Accordingly, this could justify NOM is adequate in most high-grade BHSI patients.

Among previous organ-specific studies, those examining high-grade BHI, reported the use of initial NOM in 78%(63/81) of patients with a failure of 3.7%(3/81) [13]. In the present study, 91(60%) patients had high-grade BHI, of which 80(88%) were managed nonoperatively, with 3.7%(3/80) failures. In terms of splenic trauma, one study [10] reported on 324(56%) patients had high-grade BSI, of which 258(79%) were managed nonoperatively, with 18(7%) failures. In present study, 59(74%) patients had high-grade BSI, of which 42(71%) were managed nonoperatively, with 7.1%(3/42) failures. Therefore, compared with previous studies analyzing high-grade injuries in a single specific organ [10, 13], our study showed similar results. It may be attributed to the standardized protocol followed at our institute, which emphasizes the early introduction of angioembolization for BHSI, and a dedicated radiology team [21, 22].

Most prior studies concluded that the main reason for the failure of NOM is the hemodynamic instability, whereas this observation was contradicted by Mitsusada et al. [23]. Various predictors of NOM failure have been documented in the literatures [2, 9, 13, 24–26]. Literature review of Bhangu et al. [24] reported AAST grades 4–5, the presence of moderate or large haemoperitoneum, increasing ISS, and increasing age were significantly associated with increased risk factor of NON failure in BSI, which led to significantly longer ICU and overall lengths of stay. Hashemzadeh et al. [25] suggested age, female gender and ISS were significant predictors of NOM failure in BHSI. In another study, Olthof et al. [26] reported age ≥ 40 years, ISS ≥ 25, splenic injury grade ≥ 3 are prognostic factors of NOM failure in BSI. In current study, lower level of hemoglobin, longer ICU LOS, and higher number of hospitalization transfusions were significant risk factors in those patients for whom NOM failed. These observations were similar with previous published studies [2, 9, 24, 27]. Robinson 3ed et al. [9] reported blood transfusion is a predictor of mortality, hospital LOS and NOM failure in BHSI. Additionally, Sartorelli et al. [27] proposed that the failure rate is higher in patients who received more than 4U of blood. In our study, in terms of the overall transfusions in ER and during hospitalization, the overall mean transfusion amounts in the s-NOM and initial NOM groups were within the 4U limit, which was in agreement with the values reported previously [27]. Further prospective study of transfusion practices in treatment algorithms of BHSI is warranted.

Another factor of NOM failure is a concomitant BHSI. In a study of Sharma et al. [28] found a higher failure rate (14.3%) than isolated liver (1.5%) or spleen (5.6%) injury. However, it was contradicted by Robinson III et al. [9]. In our study, there were only 6 patients with combined high-grade BHSI, so it is difficult to compare significance.

A potential drawback of NOM is that hollow viscus injuries are overlooked. Swaid et al. [29] reported a hollow viscus injury rate of 1.5% in a BAT with neither splenic nor hepatic injuries, 3.1% with isolated BSI, 3.1% with isolated BHI, and 6.7% with concomitant BHSI, respectively. Miller et al. [30] found an associated intra-abdominal injury rate of 5% in a NOM liver group and 1.7% in a NOM spleen group, and a missed injury rate of 2.3% and 0%, respectively. On the other hand, the reported rate of hollow organ injury is approximately 0.3% of 227972 BAT admissions with an approximately 0.6-0.8% missed injury rate in patients selected for NOM [27, 31, 32]. Thus, hollow viscus injury is not unusual in combined BHSI. Although the overall incidence of missed injury is relatively low, we should not abandon the suspicion of peritonitis in every BAT patient. In our series, there was no missed injury in initial NOM group.

Multiple studies have documented that successful NOM not only can increase organ salvage rates, but also can decrease blood transfusions requirements, hospital stays, nontherapeutic laparotomy rates, septic complications, and mortality rates [4, 5]. Studies conducted by Schnuriger et al. [13] and Velmahos et al. [15] reported complications rates of approximately 17%-20% in high-grade BHSI with NOM. On the other hand, in a collective review of 1489 non-therapeutic laparotomies, the complication rate was 14.6% [33]. Our data showed that the morbidities of s-NOM (6.8%) and initial NOM (11.4%) were lower than previous studies [13, 15, 33]. Although our numbers were low, they lend further support to the contention that the complication rate is acceptable to justify this form of therapy.

Of the two mortality cases in f-NOM group, one (ISS:34) died of concomitant severe lung contusion and the other one (ISS:38) died of coexistent liver cirrhosis coagulopathy. Fang et al. [34] considers that cirrhosis is a contraindication for NOM in BSI and suggested early surgery for these patients. Another study of Schnuriger et al. [13] suggested that concomitant injuries, especially extraabdominal lesions, are a major determinant of outcome in patients with high-grade BHI and should be consulted early by trauma surgeon. When NOM for BHSI is often advocated, we should not forget that safe NOM requires adherence to cardinal surgical principles and fastidious clinical decision-making.

The present study had two limitations; one was we put discussions of BHI and BSI together and another was the lower number of cases included in the f-NOM group. Hence, it may not be an accurate reflection of the true results of the applicability of NOM to isolated hepatic or splenic injuries. Despite these limitations, our results provided valid information on the applicability of NOM to high-grade BHSI as the data of the study was collected prospectively with strict protocols.

## Conclusions

Parallel to the rapid growth of economics in Taiwan, motor vehicles accidents will continue to contribute significantly to the high-grade BHSI. Our study shows lower morbidities in successful NOM justify further attempts for NOM in high-grade BHSI in selected patients aiming at formulating a specific standardized diagnostic/management algorithm. With the incorporation of a protocol, 95% of hemodynamically stable patients with high-grade BHSI can be managed safely with NOM. This study can help emergency practitioners and trauma surgeons recognize and introduce the practice.

## Abbreviations

NOM: Non-operative management
BHSI: Blunt hepatic and/or splenic injuries
OP: Operation
LOS: Length of stay
ICU: Intensive care unit
BAT: Blunt abdominal trauma
CT: Computed tomography
CEUS: Contrast-enhanced ultrasound
ER: Emergency room
SBP: Systolic blood pressure
HR: Heart rate
RR: Respiratory rate
GCS: Glasgow coma scale
ISS: Injury severity score
BHI: Blunt hepatic injuries
BSI: Blunt splenic injuries
BT: Blood transfusion
OR: Operation room.
